# Diabetes and all-cause mortality in hemodialysis: A comparative survival analysis with complementary machine-learning methods

**DOI:** 10.1371/journal.pone.0349639

**Published:** 2026-11-04

**Authors:** Berrak Itır Aylı, Çiğdem Ikhlef, Ebru Gök Oğuz, Nüket Paksoy

**Affiliations:** 1 Faculty of Life Sciences, University of Westminster, London, United Kingdom; 2 Department of Nephrology, Etlik City Hospital, Ankara, Türkiye; 3 Department of Nephrology, University of Health Sciences, Etlik City Hospital, Ankara, Türkiye; 4 Department of Public Health, Faculty of Medicine Hacettepe University, Ankara, Türkiye; National Healthcare Group, SINGAPORE

## Abstract

**Background:**

Diabetes mellitus is the leading cause of end-stage renal disease and confers excess mortality in patients on hemodialysis. However, which diabetes-specific clinical parameters best predict survival remains insufficiently explored.

**Methods:**

This retrospective cohort study followed 252 patients receiving maintenance hemodialysis (126 with diabetes, 126 without) from three centres in Ankara, Turkey, over 68 months (January 2020–September 2025; 66 deaths). Mortality predictors were assessed using Cox proportional hazards models, propensity score–weighted regression, and gradient-boosted machine-learning survival analysis. Eleven diabetes-specific parameters were examined within the diabetes subgroup.

**Results:**

Diabetes was associated with reduced survival (log-rank p = 0.038) and a 68% higher mortality hazard (HR 1.68, 95% CI 1.02–2.75). After multivariable adjustment, the association was attenuated but directionally consistent across all specifications (HR 1.52–1.66). Within the diabetes subgroup, HbA1c was the strongest modifiable predictor (adjusted HR 1.43 per 1%, 95% CI 1.17–1.76, p < 0.001), improving model discrimination (C-index 0.679 vs. 0.652). No other diabetes-specific parameter, including retinopathy, diabetic foot, and diabetes duration, independently predicted mortality.

**Conclusion:**

In this Turkish hemodialysis cohort, diabetes showed a consistent adverse trend toward higher mortality. HbA1c was the dominant modifiable predictor, supporting the integration of structured glycaemic monitoring into routine hemodialysis care.

## Introduction

Chronic kidney disease (CKD) affects over 850 million people globally, with 3.57 million receiving dialysis [1,2]. Hemodialysis (HD) patients face substantially reduced life expectancy, with five-year survival around 52% [3]. Cardiovascular complications and infections are among the most common causes of mortality in patients on HD globally and in Türkiye [4,5].

Diabetes mellitus is another global public health problem; it is the leading global cause of end-stage renal disease (ESRD), accounting for 31.1% of incident cases in 2015 [6]. CKD is 2–5 times more common in patients with diabetes compared to those without, and ESRD is 10 times more common in people with diabetes compared to the rest of the population [7,8]. Furthermore, people with diabetes on HD experience disproportionately higher morbidity and mortality compared to their counterparts without [9,10].

The reasons behind the excess mortality in dialysis patients with diabetes are multifactorial. Diabetes contributes to poor glycaemic control, accelerated atherosclerosis, endothelial dysfunction, and chronic inflammation, causing significant complications in several other organs all of which predispose to cardiovascular disease; the leading cause of death in dialysis [10,11]. It also increases the risk of infection through impaired immune function and vascular complications, whereas dialysis-specific factors such as vascular access type, adequacy of dialysis, residual renal function, and nutritional/inflammatory markers play additional critical roles in survival outcomes [12,13]. However, how the presence of diabetes modifies the prognostic significance of these factors remains insufficiently explored.

Therefore, the present study investigates predictors of all-cause mortality in a cohort of patients on HD with an emphasis on the role of diabetes mellitus, HbA1c and diabetic complications. In addition to the conventional Cox proportional hazards models, advanced analytic strategies, including propensity score weighting, subgroup analyses, and machine-learning survival models for survival prediction, are applied. By combining traditional survival analyses with modern predictive modelling methods like machine learning, this study aims to provide a more nuanced understanding of risk stratification in HD populations with DM and contribute to creating detailed predictive models that can be utilised to develop targeted interventions [14].

## Methods

### Study design and setting

This was a retrospective cohort study conducted across three hemodialysis centres in Ankara, Türkiye. The study baseline was January 2020 and patients were followed until 30 September 2025 (68-month observation period). Clinical and laboratory data were collected retrospectively from electronic medical records throughout October 2025. The study protocol was approved by the Etlik City Hospital Ethics Committee (AESH-BADEK-2025–0257).

### Participants and sampling

The source population comprised all adult patients (age ≥ 18 years) receiving maintenance hemodialysis at the three participating centres as of January 2020. This date served as the study baseline (time zero), at which all clinical and laboratory variables were ascertained. The authors collected the data throughout October 2025 from the electronic record systems of the participating institutions; all data were collected without identifying information. The cohort therefore represents a prevalent hemodialysis population under active treatment at study entry.

#### Diabetes group.

All patients with a documented diagnosis of diabetes mellitus (type 1 or type 2) who were receiving maintenance hemodialysis at the participating centres in January 2020 were evaluated for inclusion (n = 149). Of these, 23 were excluded: 4 because of a change in renal replacement modality during follow-up (transplantation or transfer to peritoneal dialysis) and 19 because of loss to follow-up (transfer to a non-participating centre). The final diabetes group comprised 126 patients.

#### Non-diabetes control group.

From the remaining patients on hemodialysis without diabetes at the same centres, 126 were randomly selected using simple random sampling to serve as the comparator group, yielding a 1:1 ratio. This 1:1 sampling ratio was chosen to ensure balanced group sizes for survival modelling while preserving the representativeness of the non-diabetic HD population at these centres. No matching on age, sex, or other covariates was performed; between-group differences were addressed through multivariable adjustment and propensity score weighting.

### Follow-up and outcomes

Patients were followed from baseline (January 2020) until death from any cause, or administrative censoring on 30 September 2025, whichever occurred first. The primary outcome was all-cause mortality. Vital status was ascertained from medical records and centre registries at each participating facility. Survival time was calculated as the interval from baseline to the date of death or censoring, expressed in months.

### Data collection

All data were extracted from patient medical records at baseline (January 2020). The following variables were collected:

- Demographic and socioeconomic: age, sex, educational attainment (five categories: illiterate, literate, primary school, high school, university).
- Clinical and comorbidity: primary cause of kidney disease, diabetes mellitus status, hypertension, coronary artery disease/heart failure, stroke, chronic obstructive pulmonary disease, and malignancy.
- Dialysis-related: dialysis vintage (months), vascular access type (arteriovenous fistula, catheter), number of AVF operations, dialysis frequency (sessions per week), ultrafiltration volume, Kt/V, urea reduction ratio (URR), residual urine output (present/absent), and dialysis anticoagulation type.
- Anthropometric and laboratory: height, weight, body mass index, systolic and diastolic blood pressure, serum albumin, hemoglobin, fasting glucose, and HbA1c (measured in patients with diabetes only).
- Medications: erythropoiesis-stimulating agents, statins, anticoagulants, cinacalcet, sevelamer, calcium-based phosphate binders, beta-blockers, ACE inhibitors/ARBs, aspirin, and calcium channel blockers.
- Diabetes-specific parameters (DM group only): diabetes duration, diabetes type, diabetic retinopathy, vision grade, diabetic foot history, prior amputation, glucometer use, home glucose monitoring, symptomatic hypoglycaemia history, hyperglycaemic coma history, diabetic diet adherence, and diabetes follow-up specialty.
- Composite Nutritional Index: The Geriatric Nutritional Risk Index (GNRI) was calculated as (14.89 × albumin [g/dL]) + (41.7 × [body weight/ideal body weight]) using the Lorentz formula for ideal weight, with the weight ratio capped at 1.0.

### Statistical analysis

#### Descriptive statistics.

Baseline characteristics were summarised as mean ± standard deviation for continuous variables and as frequencies with percentages for categorical variables. Between-group differences were assessed using independent-samples t-tests or Mann–Whitney U tests (based on Shapiro–Wilk normality testing) for continuous variables and chi-squared tests for categorical variables.

#### Survival analysis.

The Kaplan–Meier method was used to estimate observed survival, with between-group comparisons made using the log-rank test. Univariable Cox proportional hazards regression was performed for all collected variables; those with p < 0.10 or established clinical relevance were considered for multivariable modelling. The prespecified core multivariable model included age, sex, diabetes status, serum albumin, Kt/V, and residual urine output. The proportional hazards assumption was assessed using Schoenfeld residuals; where violations were detected, stratified Cox models were fitted with the offending variable as the stratum. Results are reported as hazard ratios (HR) with 95% confidence intervals. Concordance indices (C-index) are reported for discrimination.

#### Propensity score weighting.

To strengthen causal inference regarding the diabetes–mortality association, a propensity score for diabetes was estimated using logistic regression with 12 baseline covariates (age, sex, hypertension, coronary artery disease, stroke, albumin, Kt/V, hemoglobin, BMI, systolic blood pressure, dialysis vintage, and residual urine). Stabilised inverse probability of treatment weights (SIPTW) were computed, with extreme weights truncated at the 99th percentile and propensity scores trimmed at the 1st and 99th percentiles. A weighted Cox model with robust standard errors was then fitted with diabetes as the sole exposure.

#### Machine-learning survival model.

As a complementary predictive approach, a gradient-boosted survival analysis model was fitted using 11 clinical variables (age, sex, diabetes, albumin, Kt/V, residual urine, hemoglobin, BMI, dialysis vintage, systolic blood pressure, and GNRI). Model performance was evaluated using five-fold stratified cross-validation, with Harrell’s concordance index as the primary metric. Variable importance was derived from the fitted model. Calibration was assessed by comparing predicted and observed mortality across decile groups at 36 and 60 months. Clinical utility was evaluated using decision curve analysis at the 60-month horizon. The machine-learning model was applied as a complementary tool for risk stratification and variable importance ranking, rather than as a replacement for regression-based inference.

#### Subgroup and interaction analyses.

The diabetes effect was examined across prespecified subgroups defined by sex and age tertiles. Multiplicative interaction terms (age × DM, sex × DM) were added to the core Cox model to test for effect modification.

#### Sensitivity analyses.

Six sensitivity analyses were performed to evaluate the robustness of the diabetes–mortality association: (i) exclusion of deaths occurring within 6 months of baseline; (ii) exclusion of deaths within 12 months; (iii) replacement of albumin with the GNRI; (iv) additional adjustment for vascular access type; (v) propensity score weighting; and (vi) stratified Cox modelling. Diabetes-specific variables (retinopathy, diabetic foot, amputation, HbA1c, diabetes duration) were analysed in univariable and multivariable models within the diabetes subgroup.

#### Software.

All analyses were conducted using Python 3.12 with the lifelines (v0.29), scikit-survival (v0.22), scikit-learn (v1.5), and scipy (v1.14) packages. Statistical significance was defined as two-sided p < 0.05.

## Results

### Baseline characteristics

A total of 457 patients receiving maintenance hemodialysis at the three participating centres were screened. From the population with diabetes, 149 patients were evaluated for inclusion, of whom 23 were excluded (4 changed renal replacement modality; 19 were lost to follow-up), yielding 126 in the diabetes group. From the remaining non-diabetic patients, 126 were selected by simple random sampling. Of the 252 patients included in the final analysis, 126 had diabetes mellitus (DM) and 126 were non-diabetic controls randomly selected from the same three hemodialysis centres in Ankara. The overall cohort had a mean age of 61.8 ± 13.7 years, and 93 (36.9%) were female. During a median follow-up of 68 months (January 2020 – September 2025), 66 deaths were recorded: 40 (31.7%) in the DM group and 26 (20.6%) in the non-DM group (relative risk 1.54, 95% CI 1.00–2.36, p = 0.045) (Table 1).

**Table 1:** Baseline characteristics of the study population by diabetes status.

Variable	Non-DM (n = 126)	DM (n = 126)	p-value
**Continuous variables (Mean ± SD)**			
**Age (years)**	59.36 ± 15.48	64.18 ± 11.22	0.0063
**Systolic BP (mmHg)**	118.12 ± 23.14	130.47 ± 20.61	<0.0001
**Diastolic BP (mmHg)**	74.51 ± 14.50	70.79 ± 10.85	0.0248
**Ultrafiltration (L)**	2.97 ± 0.88	2.82 ± 1.19	0.0450
**Kt/V**	1.65 ± 0.32	1.59 ± 0.33	0.0887
**URR (%)**	74.57 ± 6.39	71.99 ± 6.28	0.0039
**Albumin (g/dL)**	3.90 ± 0.42	3.68 ± 0.42	<0.0001
**Hemoglobin (g/dL)**	11.14 ± 1.82	10.88 ± 1.47	0.1050
**Body Mass Index (kg/m²)**	23.56 ± 4.36	27.16 ± 5.21	<0.0001
**Glucose (mg/dL)**	101.96 ± 26.17	181.17 ± 71.83	<0.0001
**HbA1c (% - mmol/mol)**	NA	7.34 ± 1.44 (57 ± 8)	NA
**Height (cm)**	167.0 ± 10.0	164.7 ± 8.5	<0.0001
**Weight (kg)**	66.33 ± 15.76	73.61 ± 15.12	0.0003
**AVF operations (n)**	1.41 ± 1.17	1.48 ± 0.99	0.3097
**Dialysis vintage (months)**	100.80 ± 71.59	73.02 ± 39.72	0.0006
**GNRI**	98.0 ± 7.2	95.6 ± 6.8	0.007
**Categorical variables (n, %)**			
**Deaths**	26 (20.6%)	40 (31.7%)	0.045
**Gender (female/male)**	40 (31.7%) / 86 (68.3%)	53 (42.1%) / 73 (57.9%)	0.1172
**Education level**			0.0004
Illiterate	10 (7.9%)	11 (8.7%)	
Literate	22 (17.5%)	53 (42.1%)	
Primary	32 (25.4%)	17 (13.5%)	
High school	37 (29.4%)	30 (23.8%)	
University	25 (19.8%)	15 (11.9%)	
**Tobacco use**			0.4361
None	59 (46.8%)	63 (50.0%)	
Ex-smoker	28 (22.2%)	20 (15.9%)	
Active	39 (31.0%)	43 (34.1%)	
**Primary CKD reason**			<0.0001
DM	0 (0.0%)	112 (88.9%)	
HT	26 (20.6%)	7 (5.6%)	
ADPKD	6 (4.8%)	2 (1.6%)	
GN	10 (7.9%)	2 (1.6%)	
Other	84 (66.7%)	3 (2.4%)	
**Hypertension (No / Yes)**	61 (48.4%) / 65 (51.6%)	41 (32.5%) / 85 (67.5%)	0.0148
**COPD (No / Yes)**	118 (93.7%) / 8 (6.3%)	118 (93.7%) / 8 (6.3%)	1.0000
**CAD (No / Yes)**	100 (79.4%) / 26 (20.6%)	84 (66.7%) / 42 (33.3%)	0.0333
**Stroke (No / Yes)**	124 (98.4%) / 2 (1.6%)	117 (92.9%) / 9 (7.1%)	0.0597
**Malignancy (No / Yes)**	119 (94.4%) / 7 (5.6%)	124 (98.4%) / 2 (1.6%)	0.1718
**Residual urine (No / Yes)**	44 (34.9%) / 82 (65.1%)	73 (57.9%) / 53 (42.1%)	0.0004
**ESA use (No / Yes)**	49 (38.9%) / 77 (61.1%)	26 (20.6%) / 100 (79.4%)	0.0024
**Anticoagulation (No / Yes)**	113 (89.7%) / 13 (10.3%)	97 (77.0%) / 29 (23.0%)	0.0112
**Sessions/week (2 / 3)**	9 (7.1%) / 117 (92.9%)	19 (15.1%) / 107 (84.9%)	0.0712
**Statin (No / Yes)**	111 (88.1%) / 14 (11.1%)	108 (85.7%) / 18 (14.3%)	0.5867
**Cinacalcet (No / Yes)**	104 (82.5%) / 20 (15.9%)	120 (95.2%) / 6 (4.8%)	0.0062
**Sevelamer (No / Yes)**	93 (73.8%) / 33 (26.2%)	101 (80.2%) / 25 (19.8%)	0.2948
**Calcium binder (No / Yes)**	44 (34.9%) / 82 (65.1%)	69 (54.8%) / 57 (45.2%)	0.0024
**β-blocker (No / Yes)**	85 (67.5%) / 41 (32.5%)	74 (58.7%) / 52 (41.3%)	0.1917
**ACEI/ARB (No / Yes)**	101 (80.2%) / 25 (19.8%)	110 (87.3%) / 16 (12.7%)	0.1721
**ASA (No / Yes)**	66 (52.4%) / 60 (47.6%)	46 (36.5%) / 79 (62.7%)	0.0185
**CCB (No / Yes)**	58 (46.0%) / 67 (53.2%)	48 (38.1%) / 77 (61.1%)	0.2494

Values are presented as mean ± standard deviation or n (%).

DM: diabetes mellitus; BP: blood pressure; URR: urea reduction ratio; GNRI: geriatric nutritional risk index; ESA: erythropoiesis-stimulating agent; ACEI: angiotensin-converting enzyme inhibitor; ARB: angiotensin receptor blocker; CCB: calcium channel blocker.

Patients with diabetes were older on average (64.2 ± 11.2 vs. 59.4 ± 15.5 years, p = 0.006), had higher systolic blood pressure (130.5 ± 20.6 vs. 118.1 ± 23.1 mmHg, p < 0.001), higher body mass index (27.2 ± 5.2 vs. 23.6 ± 4.4 kg/m^2^, p < 0.001), and higher fasting glucose levels (181.2 ± 71.8 vs. 102.0 ± 26.2 mg/dL, p < 0.001). Serum albumin was lower in the DM group (3.68 ± 0.42 vs. 3.90 ± 0.42 g/dL, p < 0.001).

Comorbidities were more prevalent among patients with diabetes, including hypertension (67.5% vs. 51.6%, p = 0.015) and coronary artery disease (33.3% vs. 20.6%, p = 0.033). Dialysis vintage was shorter in the DM group (73.0 ± 39.7 vs. 100.8 ± 71.6 months, p = 0.001).

### Kaplan–Meier survival analysis

Kaplan–Meier analysis demonstrated reduced survival in patients with diabetes compared with patients without throughout the observation period (log-rank p = 0.038; Fig 1). At 36 months, estimated survival was 90.5% in the DM group versus 92.9% in the non-DM group; at 60 months, the estimates diverged further to 72.2% versus 86.5%. Overall cohort survival was 91.7% at 36 months and 79.4% at 60 months.

**Fig 1:**
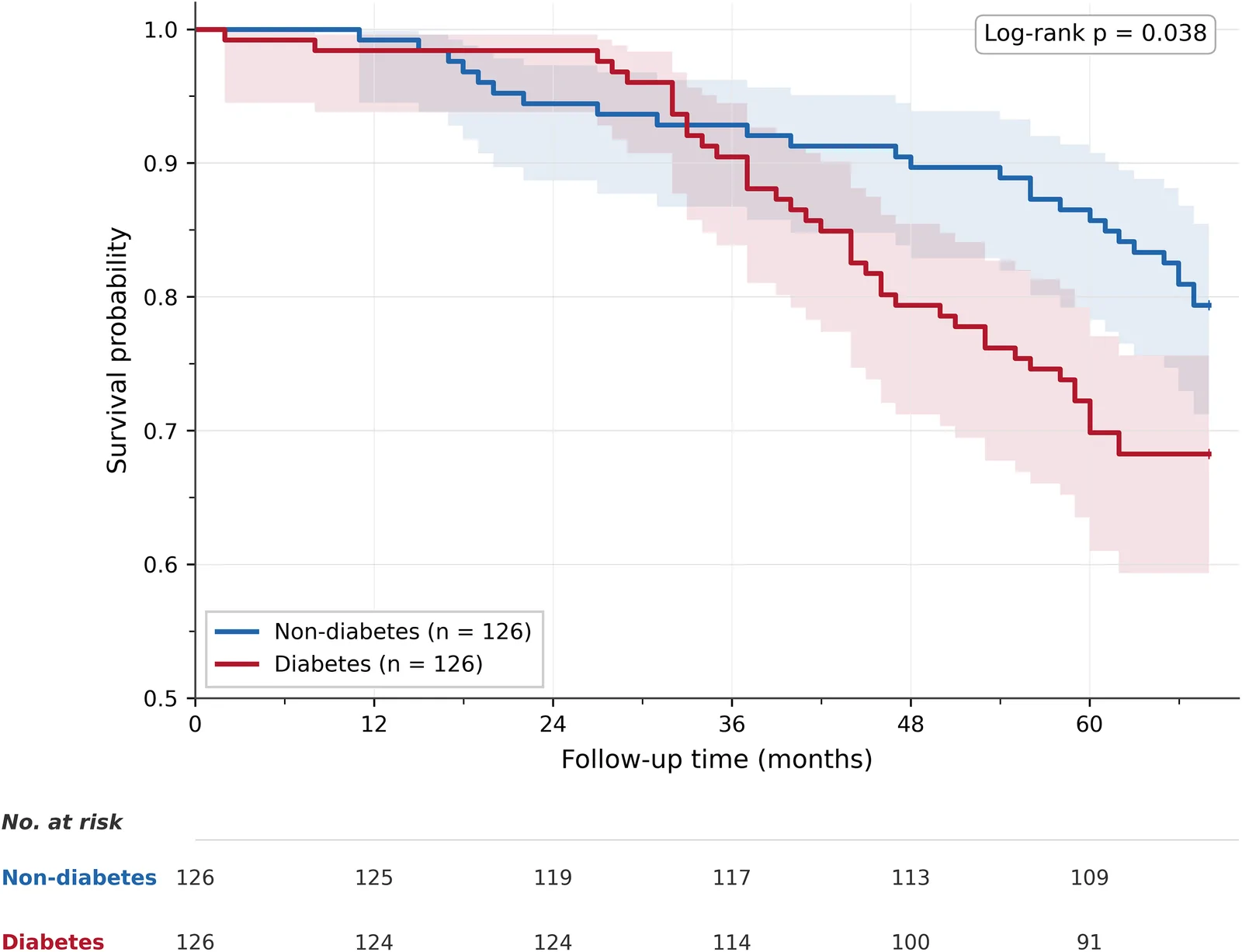
Kaplan–Meier survival curves for patients with and without diabetes, with 95% confidence intervals.

### Univariable Cox regression

In univariable Cox regression (Table 2), diabetes was associated with a 68% increase in the hazard of death (HR 1.68, 95% CI 1.02–2.75, p = 0.040). Older age was also significantly associated with mortality (HR 1.04 per year, 95% CI 1.02–1.06, p < 0.001). Thrice-weekly dialysis sessions were protective compared to twice-weekly (HR 0.43, 95% CI 0.24–0.78, p = 0.005), and higher educational attainment showed a protective association (HR 0.81 per category, 95% CI 0.67–0.99, p = 0.038). Diastolic blood pressure (HR 0.98, p = 0.076) and COPD (HR 2.03, p = 0.076) showed borderline trends. Serum albumin, hemoglobin, BMI, Kt/V, and vascular access type were not significantly associated with mortality in univariable analysis.

**Table 2:** Univariable and multivariable cox regression results.

Variable	HR	95% CI	p-value	Model
**Diabetes (DM)**	1.68	1.02–2.75	0.040	**Univariable**
**Age (per year)**	1.04	1.02–1.06	< 0.001	
**Sessions/week (3 vs 2)**	0.43	0.24–0.78	0.005	
**Education (per category ↑)**	0.81	0.67–0.99	0.038	
**Diastolic BP (per mmHg)**	0.98	0.96–1.00	0.076	
**COPD**	2.03	0.93–4.45	0.076	
**Stroke**	2.15	0.86–5.35	0.100	
**Glucose (per mg/dL)**	1.003	1.00–1.01	0.100	
**Age (per year)**	1.04	1.02–1.06	< 0.001	**Core Multivariable**
**Male sex**	1.34	0.79–2.28	0.284	
**Diabetes (DM)**	1.59	0.94–2.69	0.082	
**Albumin (g/dL)**	1.12	0.62–2.04	0.710	
**Kt/V**	1.84	0.83–4.07	0.134	
**Residual urine**	0.72	0.44–1.18	0.198	
**DM (GNRI-adjusted†)**	1.55	0.92–2.59	0.096	**Extended Multivariable**
**DM (IPTW‡)**	1.66	0.90–3.08	0.106	

HR, hazard ratio; CI, confidence interval. Variables with p < 0.10 shown for univariable cox regression.

Core model C-index = 0.652; GNRI model C-index = 0.652. †GNRI model replaces albumin with the Geriatric Nutritional Risk Index. ‡IPTW: inverse probability of treatment weighting using stabilised weights. Proportional hazards assumption tested with Schoenfeld residuals; DM violated the assumption (p < 0.001).

### Multivariable Cox regression

The prespecified core multivariable model included age, sex, diabetes, serum albumin, Kt/V, and residual urine output (Table 2). Age was the strongest independent predictor (HR 1.04 per year, 95% CI 1.02–1.06, p < 0.001). Diabetes was associated with a 59% increase in the hazard of mortality (HR 1.59, 95% CI 0.94–2.69, p = 0.082), which did not reach conventional significance at α = 0.05 after adjustment. Albumin, Kt/V, residual urine, and sex were not independently associated with mortality (all p > 0.1). The concordance index for the core model was 0.652. The proportional hazards assumption was violated for diabetes (Schoenfeld p < 0.001); a stratified Cox model confirmed robustness of all other covariate estimates.

In a propensity score–weighted analysis (Fig S1 in S1 File), using stabilised inverse probability of treatment weights derived from 12 baseline covariates (propensity score model AUC = 0.76), the hazard ratio for diabetes was 1.66 (95% CI 0.90–3.08, p = 0.106), directionally consistent with the core model and sensitivity analyses (Section 3.8).

### Hba1c and mortality in the diabetes subgroup

Within the diabetes subgroup (n = 126, 40 events), HbA1c was the strongest modifiable predictor of mortality (Table S1 in S1 File, Fig 2). Each 1% increase in HbA1c was associated with a 43% increase in the hazard of death (HR 1.43, 95% CI 1.17–1.76, p < 0.001). Age remained independently significant (HR 1.05 per year, p = 0.002), and residual urine output was protective (HR 0.43, 95% CI 0.21–0.88, p = 0.020). This model achieved a concordance index of 0.679, representing a meaningful improvement over the core model (0.652). The mean HbA1c in the diabetes group was 7.34% ± 1.44 (57 ± 8 mmol/mol).

**Fig 2:**
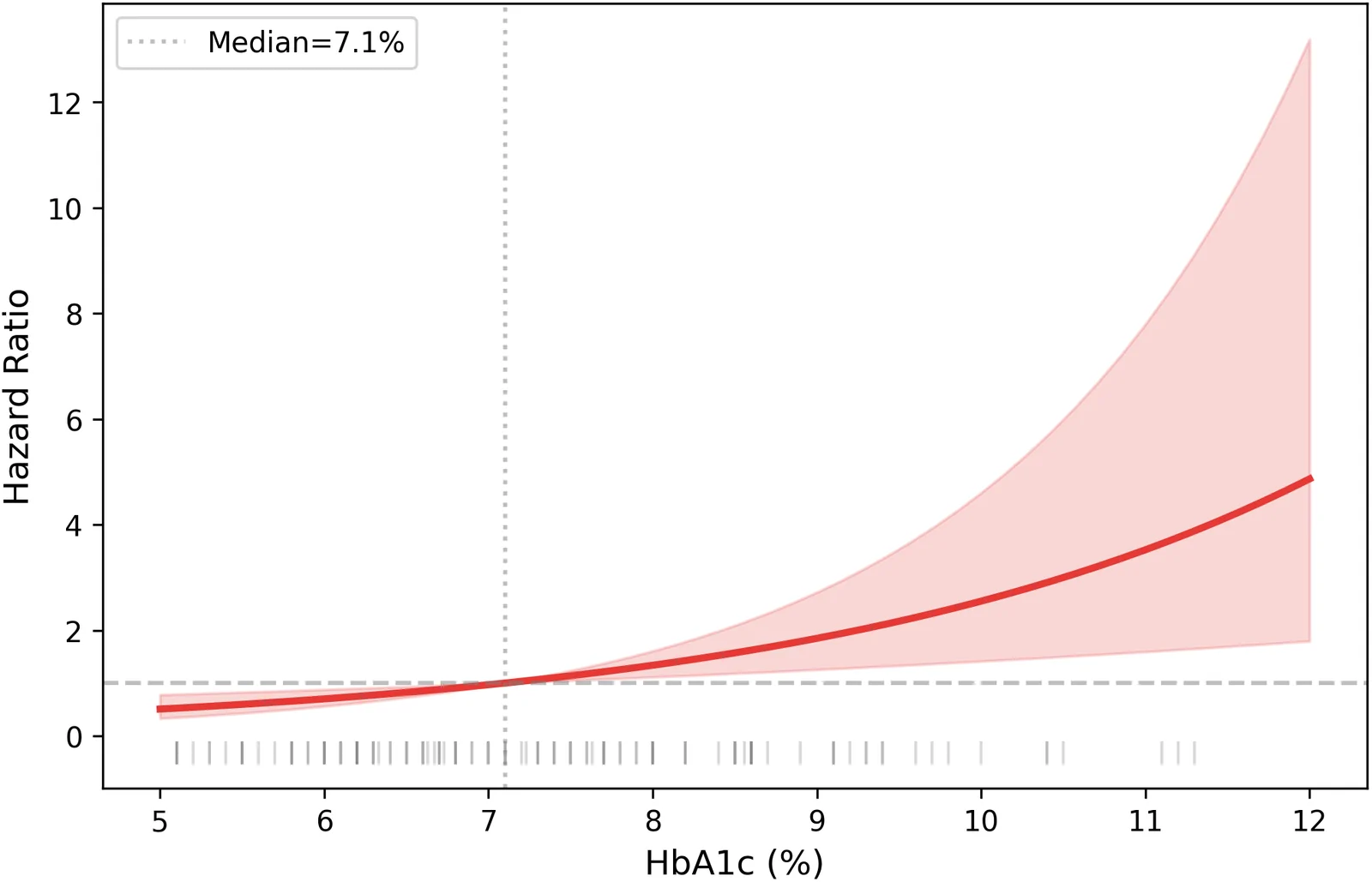
Adjusted hazard ratio for mortality as a function of HbA1c.

### Diabetes-specific clinical parameters

Among 11 diabetes-specific clinical variables tested in univariable Cox regression within the DM subgroup (Table 3), only HbA1c was significantly associated with mortality (HR 1.30 per 1%, 95% CI 1.06–1.60, p = 0.012). Diabetes duration (mean 21.2 ± 10.7 years), presence of retinopathy (85.7%), diabetic foot history (39.7%), prior amputation (15.9%), symptomatic hypoglycaemia history, hyperglycaemic coma history, adherence to a diabetic diet, home glucose monitoring, glucometer use, and visual impairment grade were all non-significant (all p > 0.1) (Fig 3).

**Table 3:** Univariable Cox regression of diabetes-specific variables (DM subgroup, n = 126).

Variable	Value (n, % or mean ± SD)*	HR	95% CI	p-value
**DM duration (per year)**	21.2 ± 9.0 years	1.01	0.97–1.04	0.705
**Retinopathy (yes vs no)**	108 (85.7%)	1.60	0.57–4.51	0.370
**Diabetic foot history**	50 (39.7%)	0.91	0.48–1.73	0.778
**Amputation history**	20 (15.9%)	1.12	0.50–2.53	0.785
**Hypoglycaemia history**	48 (38.1%)	0.57	0.28–1.14	0.111
**Hyperglycaemic coma history**	50 (39.7%)	1.24	0.66–2.31	0.500
**Diabetic diet adherence**	69 (54.8%)	1.03	0.55–1.93	0.918
**Daily home glucose monitoring**	57 (45.2%)	0.89	0.48–1.67	0.718
**Glucometer use**	117 (92.9%)	1.09	0.33–3.52	0.890
**HbA1c (per 1%)**	7.34 ± 1.44%	1.30	1.06–1.60	0.012
**Vision grade (per level)**	normal 23 (18.3%), decreased 98 (77.8%), none/blind 5 (4.0%)	1.04	0.56–1.91	0.906

*Values show the number/percentage of positive patient.

**Fig 3:**
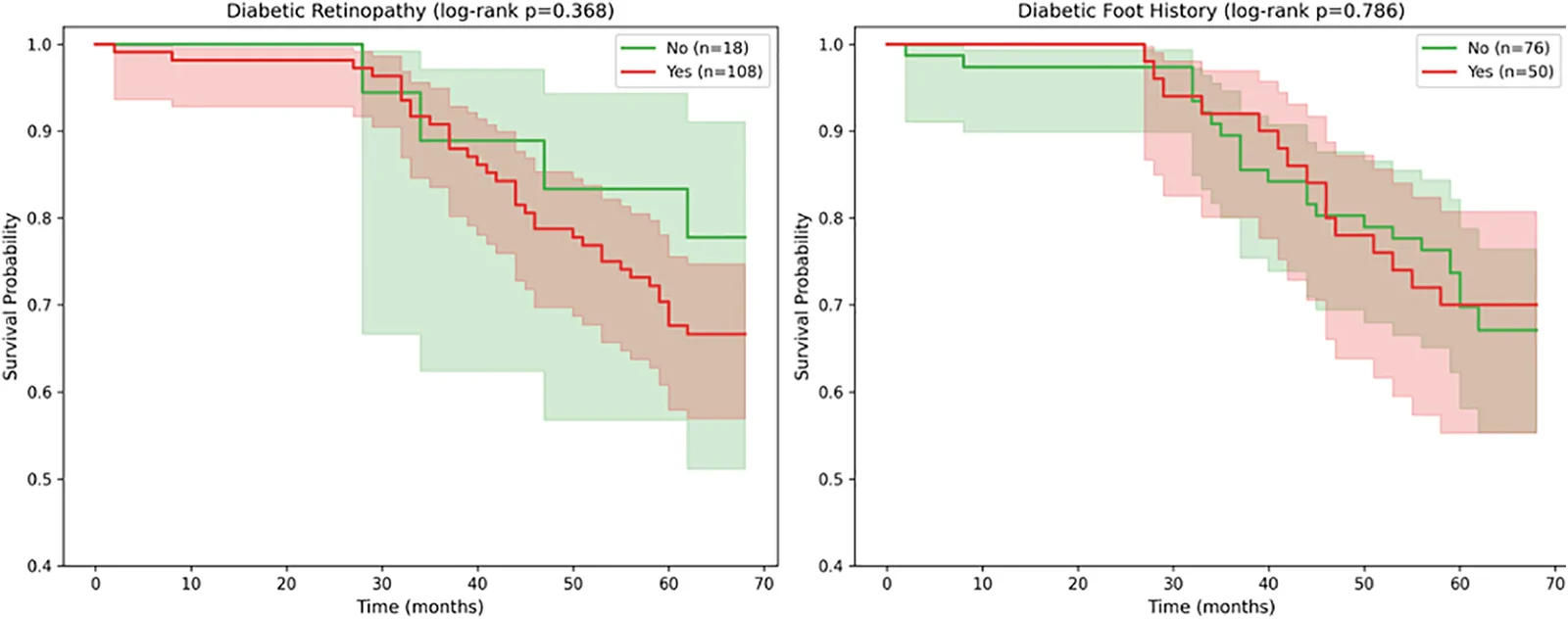
Kaplan–Meier survival within the DM subgroup by (A) retinopathy status and (B) diabetic foot history.

In a multivariable model incorporating age, sex, albumin, HbA1c, retinopathy, diabetes duration, and diabetic foot, only age (HR 1.06, p = 0.002) and HbA1c (HR 1.36, 95% CI 1.11–1.67, p = 0.003) retained significance (model C-index = 0.671).

### Subgroup and interaction analyses

No statistically significant interaction was detected between diabetes and age (interaction p = 0.539) or between diabetes and sex (interaction p = 0.359). However, stratified analyses revealed a notable sex-specific pattern (Table S2 in S1 File): the diabetes-associated mortality hazard was statistically significant only among male patients (HR 2.12, 95% CI 1.12–4.02, p = 0.021), whereas no effect was observed among females (HR 1.00, 95% CI 0.41–2.43, p = 0.994). Across age tertiles, the DM effect was directionally consistent (HR 1.48–1.99) but did not reach significance in any stratum, likely reflecting reduced statistical power after stratification.

### Machine-learning survival model

A gradient-boosted survival model was fitted using 11 clinical variables and evaluated with five-fold stratified cross-validation. The cross-validated concordance index was 0.660 ± 0.094, comparable to the Cox core model (0.652) (Table S3 in S1 File). Variable importance ranked dialysis vintage, age, and BMI highest, with diabetes ranking ninth.

Predicted survival curves from the ML model closely tracked Kaplan–Meier estimates for both groups throughout follow-up (Fig 4). Calibration was adequate (slopes 1.03 at 36 months, 1.25 at 60 months; Fig S2 in S1 File) and decision curve analysis confirmed positive net benefit across 5–40% threshold probabilities (Fig S3 in S1 File).

**Fig 4:**
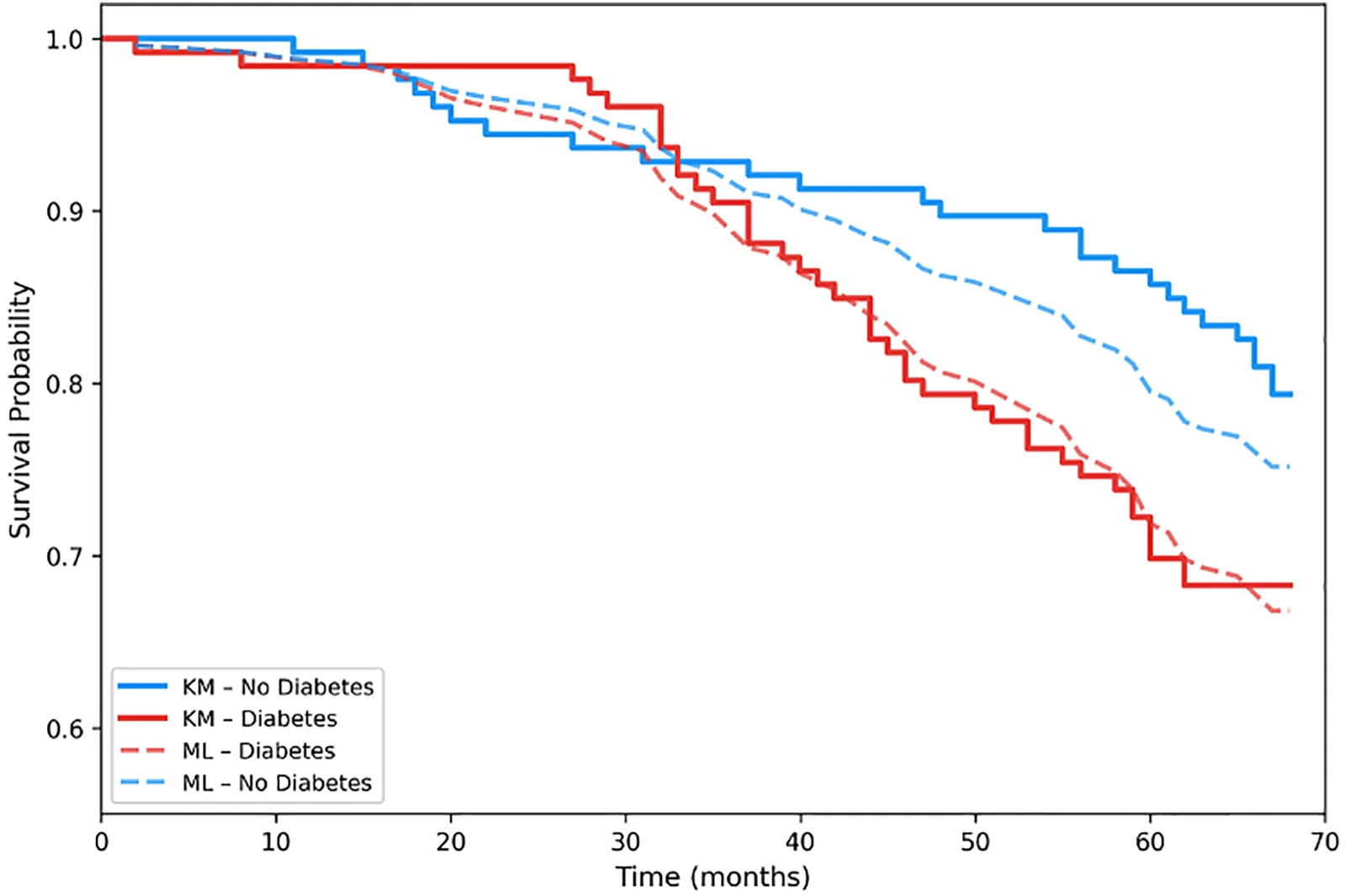
Observed (KM) vs. predicted (ML) survival curves by diabetes status.

### Sensitivity analyses

The diabetes–mortality association was robust across all sensitivity specifications (Table S4, Fig S4 in S1 File). Exclusion of early deaths (< 6 months: HR 1.57; < 12 months: HR 1.60), replacement of albumin with GNRI (HR 1.55), additional adjustment for vascular access type (HR 1.52), and propensity score weighting (HR 1.66) all yielded hazard ratios in the range of 1.52–1.66. Although no individual adjusted estimate reached conventional significance, the consistent direction and magnitude across six independent specifications suggests a clinically relevant adverse trend.

## Discussion

In this multicentre Turkish hemodialysis cohort, diabetes mellitus was associated with significantly reduced survival in Kaplan–Meier analysis (log-rank p = 0.038) and a 68% increase in the univariable mortality hazard (HR 1.68, p = 0.040). After multivariable adjustment for age, sex, albumin, Kt/V, and residual urine, the diabetes effect was attenuated but directionally consistent across all model specifications (HR range 1.52–1.66), and propensity score weighting confirmed a comparable estimate (HR 1.66). The Schoenfeld residual test revealed a violation of the proportional hazards assumption for diabetes (p < 0.001), indicating that the diabetes–mortality relationship was not constant over time. HbA1c emerged as the strongest modifiable predictor of mortality within the diabetes subgroup (HR 1.43 per 1%, p < 0.001), and a sex-stratified analysis identified a significant diabetes–mortality association among men (HR 2.12, p = 0.021) that was entirely absent in women (HR 1.00, p = 0.994). Machine-learning and classical survival models converged on the same principal predictors and produced closely aligned survival estimates.

Our univariable finding that diabetes confers excess mortality in hemodialysis is consistent with large international cohorts. The DOPPS study identified diabetes as an important predictor of adverse outcomes in patients on hemodialysis across multiple countries [15], and a Polish registry-based study found that diabetes was associated with significantly higher all-cause mortality in ESRD patients compared with the general population [10]. The attenuation of the diabetes effect after multivariable adjustment, falling to HR 1.59 (p = 0.082) in the core model, mirrors findings from studies where the independent contribution of diabetes diminished once age and comorbidity burden were accounted for [13]. This pattern suggests that diabetes operates partly through intermediate pathways (hypertension, cardiovascular disease, malnutrition) that are themselves adjusted for in multivariable models, rather than solely as an independent risk factor.

The detection of a proportional hazards violation for diabetes is a methodologically important finding. The non-proportionality implies that the relative hazard associated with diabetes changes over time, likely reflecting an initially stronger effect that attenuates as the surviving population with diabetes becomes progressively selected for resilience. This phenomenon has been described in other dialysis cohorts where early mortality is disproportionately concentrated among high-risk patients [3], and it underscores the importance of routinely testing and reporting the proportional hazards assumption in observational survival studies. We addressed this through a stratified Cox model, which confirmed that the effects of other covariates were robust to the non-proportionality of diabetes.

HbA1c was the strongest modifiable predictor in the diabetes subgroup, with each 1% increase associated with a 43% increase in mortality hazard (p < 0.001). This aligns with DOPPS-based findings by Ramirez et al., who demonstrated a U-shaped relationship between HbA1c and mortality in diabetic hemodialysis patients, with excess risk when HbA1c diverged from the 7–7.9% range [16]. Hoshino et al. confirmed these U-shaped patterns across different DOPPS countries [17]. The monotonic increase in hazard we observed above HbA1c 7.1% is consistent with the right arm of this U-shaped curve and likely reflects our limited ability to detect the left-arm risk among the small number of patients with very low HbA1c. Adding HbA1c to the Cox model improved the C-index from 0.652 to 0.679, a clinically meaningful gain in discrimination. Current KDIGO 2024 guidelines acknowledge HbA1c measurement caveats in advanced CKD (altered erythrocyte turnover, carbamylation, ESA use) but continue to recommend its use with individualised targets [18]. Our findings reinforce HbA1c as a prognostically relevant marker in hemodialysis and support the clinical utility of systematic HbA1c monitoring in this population.

A notable finding was the sex-specific difference in the diabetes–mortality association: the effect was significant only in men (HR 2.12, p = 0.021) and absent in women (HR 1.00, p = 0.994), though the formal interaction term did not reach significance (p = 0.359). This contrasts with the NECOSAD study by Carrero et al., which found that the diabetes-associated mortality risk was actually higher in women (HR 2.93) than in men (HR 1.99) among incident dialysis patients [19]. The DOPPS also reported that the mortality risk was lower for men than for women on hemodialysis [20]. Several explanations for our discordant finding may be considered: the small number of female deaths may have limited power to detect a female-specific effect; baseline sex differences in comorbidity and treatment patterns may differ in the Turkish context; and the prevalent-cohort design may introduce differential survival selection by sex. Nevertheless, the finding warrants further investigation in larger Turkish and Middle Eastern hemodialysis registries, as sex-specific risk stratification could inform targeted prevention strategies.

Among 11 diabetes-specific clinical parameters tested, only HbA1c was significantly associated with mortality. Retinopathy, diabetic foot history, amputation, diabetes duration, and self-management variables were all non-significant. This is a clinically important negative finding: in an established hemodialysis population, the overall metabolic burden of diabetes, captured by HbA1c, appears more prognostically relevant than specific microvascular end-organ complications. This may reflect the fact that hemodialysis patients already represent a highly selected population in whom end-organ complications are apparent in some degree as 89% of the group had DM as the primary reason for ESRD. Retionpathy was also highly prevalent (85.7%) potentially limiting its discriminative ability for mortality prediction. Although diabetic foot (39.7%) and amputation (15.9%) were less prevalent, neither showed a significant association, suggesting that in this cohort HbA1c captures the prognostically relevant metabolic burden more effectively than individual end-organ complications.

The machine-learning survival model (gradient-boosted survival analysis) achieved a cross-validated C-index of 0.660 ± 0.094, comparable to the Cox core model (0.652). Variable importance rankings identified dialysis vintage, age, and BMI as the dominant predictors, with diabetes itself contributing relatively little independent information; consistent with the notion that the metabolic consequences of diabetes (captured through albumin, BMI, hemoglobin) drive mortality risk rather than diabetes status per se. The close agreement between ML-predicted and Kaplan–Meier survival curves, acceptable calibration, and positive net benefit on decision curve analysis support the validity of the predictive framework.

These findings carry several practical implications. First, HbA1c monitoring should be systematically integrated into HD quality metrics for patients with diabetes, with individualised glycaemic targets informed by the patient’s age, comorbidity, and life expectancy. Second, the sex-specific vulnerability identified in this study, if replicated, could support sex-stratified risk assessment and targeted cardiovascular prevention in male diabetic hemodialysis patients. Third, the consistency of the diabetes–mortality association across six independent analytical specifications, despite marginal statistical significance in any single model, argues for clinical attention to diabetes management within dialysis units regardless of whether conventional p-value thresholds are crossed in individual studies. Educational and self-management interventions, which have been shown to improve disease awareness in diabetic CKD populations [21], may represent an additional pathway for improving outcomes.

### Strengths and limitations

This study has several methodological strengths. The use of multiple complementary analytical approaches, including Cox regression, stratified Cox models, propensity score weighting, machine-learning survival analysis with cross-validation, and six sensitivity specifications, provides a comprehensive assessment of the diabetes–mortality relationship that goes beyond what a single model can offer. The reporting of calibration, discrimination, and decision curve analysis aligns with current recommendations for prediction model studies [14]. The inclusion of diabetes-specific clinical parameters (retinopathy, foot disease, self-management behaviours) is rarely available in hemodialysis mortality studies and adds novel information. The 68-month follow-up provides a meaningful observation window for survival analysis.

Several limitations should be acknowledged. First, the sample size of 252 patients with 66 events limits statistical power, particularly for subgroup analyses and interaction testing. Second, the design involved inclusion of all available patients with diabetes with random selection of non-diabetic controls from the same centres. Although this was addressed through multivariable adjustment and propensity score weighting, residual confounding cannot be excluded. Third, all laboratory and clinical variables were measured at a single baseline timepoint (January 2020); longitudinal changes in albumin, HbA1c, and dialysis adequacy were not captured. Fourth, cause-specific mortality data were not available, precluding competing-risk analysis for cardiovascular versus non-cardiovascular death. Fifth, inflammatory biomarkers (e.g., CRP, IL-6) were not systematically measured, which prevented assessment of the inflammation–malnutrition–atherosclerosis triad. Finally, the prevalent-cohort design means that patients who died before January 2020 were not included, introducing left-truncation bias and limiting generalisability to incident populations on hemodialysis.

Future studies should incorporate time-varying covariates, cause-specific mortality with competing-risk frameworks, inflammatory biomarkers, and prospectively evaluate whether risk-guided interventions, such as tailored glycaemic optimisation targets and sex-stratified cardiovascular prevention, improve outcomes in diabetic hemodialysis patients. Larger multicentre Turkish cohorts with national registry linkage would enable validation of the sex-specific findings reported here.

## Conclusion

In this Turkish hemodialysis cohort, diabetes mellitus was associated with reduced survival and a consistently elevated mortality hazard across multiple analytical approaches, though the adjusted effect did not reach conventional statistical significance in any individual multivariable model. HbA1c was the strongest modifiable predictor within the diabetes subgroup, with each 1% increase conferring a 43% rise in the mortality hazard and meaningfully improving model discrimination. A sex-stratified analysis revealed that the diabetes–mortality association was driven predominantly by male patients. Traditional dialysis adequacy measures and serum albumin did not retain independent prognostic significance after adjustment. Microvascular diabetic complications (retinopathy, foot disease, amputation) did not add predictive value beyond HbA1c. Machine-learning and classical survival models converged on the same predictors and produced closely calibrated survival estimates, supporting the complementary use of these approaches. These findings reinforce the imperative of integrating structured glycaemic management, anchored in systematic HbA1c monitoring with individualised targets, into routine hemodialysis care, particularly for male patients with diabetes.

## Supporting information

S1 File This document contains Supplementary tables and figures including four tables (S1–S4) presenting Cox regression, subgroup analyses, model performance, and sensitivity analyses, and four figures (S1–S4) illustrating propensity score distributions, calibration, decision curve analysis, and sensitivity analyses. (DOCX)
