# Supplementary material for: Diabetes and all-cause mortality in hemodialysis: A comparative survival analysis with complementary machine-learning methods
Source: PLoS One. 2026 Nov 4;21(11):e0349639. doi: 10.1371/journal.pone.0349639 (PMC13734485; doi:10.1371/journal.pone.0349639)
Supplement: Supplementary file 1 [file pone.0349639.s001.docx]

**Diabetes-Associated Mortality in Hemodialysis: A Comparative Survival Analysis with Complementary Machine-Learning Methods**

***Supplementary Materials***

**Table S1.** Cox Regression in the Diabetes Subgroup Including HbA1c

| **Variable** | **HR** | **95% CI** | **p-value** |
| --- | --- | --- | --- |
| **Age (per year)** | 1.05 | 1.02 – 1.09 | 0.002 |
| **Male sex** | 1.73 | 0.88 – 3.42 | 0.112 |
| **Albumin (g/dL)** | 1.02 | 0.46 – 2.27 | 0.968 |
| **Kt/V** | 2.53 | 0.86 – 7.47 | 0.092 |
| **Residual urine** | 0.43 | 0.21 – 0.88 | 0.020 |
| **HbA1c (per 1%)** | 1.43 | 1.17 – 1.76 | < 0.001 |

*Model fitted within the diabetes subgroup only (n = 126, 40 events). C-index = 0.679. HbA1c available only in diabetic patients*

**Table S2.** Subgroup Analyses: Diabetes Effect on Mortality

| **Subgroup** | **n** | **Events** | **DM HR** | **95% CI** | **p-value** |
| --- | --- | --- | --- | --- | --- |
| **Overall (adjusted)** | 252 | 66 | 1.59 | 0.94 – 2.69 | 0.082 |
| **Age – Young tertile** | 84 | 15 | 1.99 | 0.70 – 5.67 | 0.200 |
| **Age – Middle tertile** | 85 | 19 | 1.48 | 0.54 – 4.06 | 0.442 |
| **Age – Old tertile** | 83 | 32 | 1.86 | 0.86 – 4.03 | 0.116 |
| **Female** | 93 | 24 | 1.00 | 0.41 – 2.43 | 0.994 |
| **Male** | 159 | 42 | 2.12 | 1.12 – 4.02 | 0.021 |

*Each subgroup model adjusted for available covariates (age, sex, albumin as applicable). Interaction terms: age × DM p = 0.539; sex × DM p = 0.359.*

**Table S3.** Summary of Model Performance

| **Model** | **C-index** | **DM HR (95% CI)** | **DM p** |
| --- | --- | --- | --- |
| **Cox Core** | 0.652 | 1.59 (0.94 – 2.69) | 0.082 |
| **Cox GNRI-adjusted** | 0.652 | 1.55 (0.92 – 2.59) | 0.096 |
| **Cox DM subgroup + HbA1c** | 0.679 | - (subgroup only) | - |
| **IPTW-weighted Cox** | - (weighted) | 1.66 (0.90 – 3.08) | 0.106 |
| **ML Gradient Boosting (5-fold CV)** | 0.660 ± 0.094 | Non-parametric | - |

*C-index = Harrell's concordance index. CV = cross-validation. IPTW = inverse probability of treatment weighting. The Cox DM + HbA1c model was fitted within the diabetes subgroup only (n = 126).*

**Table S4.** Sensitivity Analyses for the Diabetes–Mortality Association

| **Model** | **DM HR** | **95% CI** | **p-value** | **Modification** |
| --- | --- | --- | --- | --- |
| **Core model (reference)** | 1.59 | 0.94 – 2.69 | 0.082 | — |
| **Exclude deaths < 6 months** | 1.57 | 0.92 – 2.65 | 0.096 | 1 early event removed |
| **Exclude deaths < 12 months** | 1.60 | 0.94 – 2.74 | 0.084 | 3 early events removed |
| **GNRI-adjusted** | 1.55 | 0.92 – 2.59 | 0.096 | GNRI replaces albumin |
| **Vascular access–adjusted** | 1.52 | 0.89 – 2.58 | 0.125 | Adds access type |
| **IPTW-weighted** | 1.66 | 0.90 – 3.08 | 0.106 | Propensity score–weighted |

*All models adjusted for age, sex, and dialysis adequacy unless otherwise specified.*

**Figure S1.** Propensity score distribution by diabetes status

**Figure S2.** Calibration plots at 36 and 60 months (10 decile groups with calibration slope

**Figure S3.** Decision curve analysis for 60-month mortality prediction

**Figure S4.** Sensitivity analyses forest plot showing DM hazard ratios across model specifications
